# Low‐intensity ultrasound induces angiogenesis by activating endothelial integrin signaling in male mice

**DOI:** 10.14814/phy2.70718

**Published:** 2026-06-26

**Authors:** Yoshitsugu Kojima, Fumitaka Mizuki, Ki‐ichiro Kawano, Yukihito Higashi

**Affiliations:** ^1^ Department of Human Genetics, Research Institute for Radiation Biology and Medicine Hiroshima University Hiroshima Japan; ^2^ Department of Clinical Pharmacology Research Laboratory Yokohama University of Pharmacy Yokohama Japan; ^3^ Division of Regeneration and Medicine Hiroshima University Hospital Hiroshima Japan

**Keywords:** angiogenesis, integrin signaling, ischemia, ultrasound

## Abstract

Peripheral arterial disease is a medical condition caused by blockages of arteries that provide blood flow to the arms or legs. Pulsed ultrasound (PUS) has been widely used for some therapies such as bone fracture healing. We hypothesized that PUS also affects angiogenesis for ischemia therapy. This study was performed to elucidate the mechanism of PUS therapeutic angiogenesis and evaluate PUS efficacy. Hindlimb ischemic model mice were applied PUS irradiation for 20 min/day for 2 weeks. In consequence, the blood flow rates in hindlimb ischemic model mice treated with PUS improved compared with those in untreated mice. Then we performed a tube formation assay using a human umbilical vein endothelial cell (HUVEC) under conditions of PUS exposure. To elucidate the signal transduction mechanism initiated by PUS stimulation in HUVECs, we evaluated phosphorylation profiles with antibody array analysis. PUS irradiation enhanced tube formation in HUVECs and upregulated phosphorylation of extracellular signal‐regulated kinase (ERK) 1/2 through the integrin pathway. Moreover, the blood flow recovery in hindlimb ischemic model mice treated with PUS was completely inhibited in mice administered an anti‐integrin blocking antibody. These findings suggested that PUS demonstrated angiogenic effects through the integrin pathway.

## 1 INTRODUCTION

Peripheral arterial disease (PAD) is a primary manifestation of atherosclerosis and is associated with increased cardiovascular morbidity and mortality (Criqui et al., 1992; Leng et al., 1996; Newman et al., 1999; Zheng et al., 1997). Several risk factors including diabetes mellitus, hypertension, dyslipidemia, aging, smoking, and obesity are involved in the development and maintenance of PAD (Adler et al., 2002; Murabito et al., 2002). Treatment options for ischemic limb revascularization are also performed, including exercise, surgical bypass, percutaneous transluminal angioplasty, and pharmacological therapy (Jude et al., 2010). Patients who are unresponsive to these treatments may require amputation (Norgren et al., 2007). The clinical benefits of cell therapy have been studied in trials (Idei et al., 2011). A novel noninvasive treatment strategy would be desirable as an alternative therapeutic option.

Ultrasound is a high‐frequency pressure wave transmitted into the body. Ultrasonic energy can be categorized as high intensity (5000–15,000 mW/cm^2^) or low intensity (0.5–3000 mW/cm^2^) (Khanna et al., 2009). Low‐intensity pulsed ultrasound (PUS) is used clinically for bone fracture (Cheung et al., 2011) and patella‐patella tendon junction (Lu et al., 2008) healing. Ultrasound induces various responses in certain cells, such as osteoblasts, chondrocytes, tenocytes, and fibroblasts (Chen et al., 2003; Mortimer & Dyson, 1988; Reher et al., 1998). Previous studies have reported that PUS stimulation induces the expression of osteogenesis‐related genes as well as angiogenic factors such as IL‐8, basic fibroblast growth factor, and vascular endothelial growth factor (VEGF) in osteoblasts (Reher et al., 1999). The therapeutic range of ultrasound stimulates both nitric oxide and prostaglandin E2 synthesis in human osteoblasts (Reher et al., 2002). In addition, ultrasound stimulation changed the cellular morphology and orientation, and increased extracellular matrix secretion from endothelial cells (Hsu & Huang, 2004). The effects of PUS on angiogenesis have been investigated previously, although the results are controversial (Rutten et al., 2008). Emerging studies indicate that PUS may promote angiogenesis by modulating paracrine signaling from mesenchymal stem cells, particularly through increased secretion of pro‐angiogenic factors such as IGF‐1 and VEGF. These secreted molecules have been shown to enhance endothelial cell proliferation, migration, and tube formation in vitro (Xu et al., 2025). Ultrasound exposure increases capillary density in rats with skin injury (Young & Dyson, 1990) and induces angiogenesis in a rat hindlimb ischemic model (Barzelai et al., 2006). A recent clinical study demonstrated that PUS reduced rest pain and enhanced the mobilization of circulating progenitor cells, suggesting its potential role in therapeutic angiogenesis (Mohamad Yusoff et al., 2025). In the present study, we employed a single low‐intensity pulsed ultrasound condition (30 mW/cm^2^, 20% duty cycle, 20 min/day), which has been widely used in previous in vivo studies. This standardized setting was selected to evaluate the fundamental biological response under a clinically relevant and reproducible condition. Future studies will be needed to explore the effects of varying intensities and treatment durations to better understand the dose‐dependent responses. Additionally, PUS stimulates cells such as synoviocytes and chondrocytes through integrin/focal adhesion kinase (FAK) signal transduction (Sato et al., 2014; Xia et al., 2015). Although integrin signaling mediates angiogenesis (Avraamides et al., 2008), whether integrin associates angiogenesis caused by PUS is not well understood.

The primary outcome of this study will be blood flow recovery assessed with PUS irradiation on the ischemic limb. Secondary outcomes constitute histological analyses to measure CD31 positive microvessels. The aims of the present study were to determine the molecular mechanism of PUS‐induced therapeutic angiogenesis. The results show that PUS treatment recovered blood flow in hindlimb ischemic model mice and induced integrin/FAK signal transduction in human umbilical vein endothelial cells (HUVECs).

## 2 MATERIALS AND METHODS

### 2.1 Mouse hindlimb ischemia model

Fifty 8‐week‐old male wild‐type C57BL/6J mice (20–25 g, median: 21.5 g) were purchased from Charles River Laboratories Japan Inc. (Yokohama, Japan). Male mice were exclusively used to establish the hindlimb ischemia model to minimize hormonal variability associated with the estrous cycle in females. The mice were housed 4–5 per cage under standard laboratory conditions on a 12‐h light/dark cycle and at a temperature of 25°C. The mice were allowed free access to water and food (Labo MR Stock, Nosan corp., Kanagawa, Japan) and were acclimatized for 1 week before the experiments.

#### 2.1.1 Ethical approval

All animal experiments were performed in accordance with the *Guide for Care and Use of Laboratory Animals* and were approved by the Institutional Committee of Laboratory Animal Experimentation at Hiroshima University (Hiroshima, Japan; approval no. A23‐57). All procedures were conducted following the ARRIVE (Animal Research: Reporting of in vivo Experiments) guidelines.

#### 2.1.2 Experimental groups and sugical procedure

The 20 mice were randomly assigned by bodyweight into non‐PUS irradiated (control: *n* = 10) and PUS groups (PUS: *n* = 10). Two days before the ischemic operation, the mice were anesthetized with isoflurane (Mylan Inc., Pittsburgh, PA, USA) with an animal anesthesia machine (DS Pharma Biomedical Co. Ltd., Osaka, Japan; induction: concentration = 4%, flow rate = 2.8 L/min; maintenance: concentration = 2%, flow rate = 2.8 L/min). The hair of the hindlimb was removed with depilatory cream (Reckitt Benckiser Japan Ltd., Tokyo, Japan). On day 0, unilateral hindlimb ischemia was induced in all mice. Anesthetized mice were placed on a heated pad maintained at 35°C. The right medial femoral skin was incised, and the femoral artery was ligated at 2 points with 5‐0 silk sutures (Nihon Chosen, Chiba, Japan). One point was distal to the origin of the lateral caudal femoral artery, and the other was distal to the side of the popliteal branch. The distal side of the femoral artery from the popliteal branch to the saphenous artery was stripped out. The incision wound was closed with 5‐0 silk sutures. At the end of the experimental period, the mice were sacrificed via carotid artery bleeding under inhalation anesthesia with isoflurane (Mylan Inc.).

### 2.2 Ultrasound stimulation for mice

On day 1, the mice subjected to hind limb ischemia were anesthetized and held as described above. Ultrasonic gel was put on an ultrasound transducer (Nippon Sigmax Co. Ltd., Tokyo, Japan) (Figure S1), and the transducer was placed over the skin of the ischemic area in the thigh. The mice in the PUS group were applied on medial muscles in the right hindlimb for 20 min/day for 14 days. The ultrasound exposure conditions were as follows: effective area of the transducer was 6.85 cm^2^; intensity was 30 mW/cm^2^ with a 20% duty cycle, and pulse frequency was 2.0 MHz with a 1‐kHz repeat rate. As in the PUS group, mice in the control group underwent ultrasonic gel application to their hindlimbs along with the placement of a transducer but no ultrasound transmission.

### 2.3 Blood flow measurement

The laser Doppler imaging (LDI) measurement was repeated 1, 3, 5, and 7 days after the operation. Under anesthetization, both the right and the left limbs were scanned with a laser Doppler perfusion image analyzer (MoorLDI2‐VR; Moor Instruments Ltd., Axminster, UK). The right side was the ischemic side and the left side was the nonischemic control side. Blood perfusion of the hindlimb was averaged for an anatomically defined region with image analysis according to the manufacturer's instructions (Moor Software V5.0).

### 2.4 Immunohistochemical tissue staining

The left and right medial thigh muscles were excised and frozen in isopentane prechilled with liquid nitrogen. After dissection at 5‐μm thickness, the muscle specimens were immunostained with anti‐CD31 antibody (BD Bioscience, San Jose, CA, USA; Cat. #550274) for vascular endothelial cells or with anti‐αSMA antibody (Sigma‐Aldrich Co. LLC., St. Louis, MO, USA) for vascular smooth muscle cells. The specimens were then reacted with secondary antibody, stained with DAB (Histofine Simple Stain Mouse MAX‐PO(R) reagent; Nichirei Biosciences Inc., Tokyo, Japan; Cat. #414341), and counterstained with hematoxylin for nuclei. Histopathological evaluations were performed with a light microscope (Olympus IX81; Olympus Corp., Tokyo, Japan) equipped with a UPLFLN 20X objective lens (numerical aperture 0.50, working distance 2.1 mm). Image acquisition was performed using Olympus cellSens software (version 4.4), which enabled consistent capture settings and standardized image processing across all samples. The number of DAB‐positive capillaries was counted in a blinded manner in five different microscopic fields on each specimen. The capillary‐to‐muscle fiber ratio was calculated to avoid overestimation of capillary number from muscle atrophy or underestimation from interstitial edema. Relative capillary density was calculated by dividing the capillary‐to‐muscle fiber ratio of the ischemic limb by that of the non‐ischemic limb. Antibodies used for immunohistochemical tissue staining are summarized in Table S1.

### 2.5 Anti‐β_1_ integrin‐blocking antibody study in hindlimb ischemia model mice

After hindlimb ischemic surgery, 30 mice were randomly assigned by bodyweight into three groups: PUS with control IgG (PUS+Control IgG: *n* = 10), PUS with anti‐β_1_ integrin antibody (PUS+anti‐Integrin β_1_ Ab: *n* = 10), and non‐PUS with control IgG (Non‐PUS+Control IgG: *n* = 10). Mini‐osmotic pumps were implanted subcutaneously in the backs of the animals in each group (Alzet model 1002; DURECT Corp., Cupertino, CA, USA) and contained either anti‐β_1_ integrin antibody (1 mg/mL, 100 μL) or isotype control IgG (1 mg/mL, 100 μL). Specifically, the PUS with anti‐β_1_ integrin antibody group received a pump filled with anti‐β_1_ integrin monoclonal antibody (Clone HMb1‐1; BioLegend Inc., San Diego, CA, USA; Cat. #16‐0291‐85), whereas the non‐PUS with control IgG and PUS with control IgG groups received pumps loaded with isotype control IgG (Clone HTK888; BioLegend Inc., Cat. #400902). During the implantation of the osmotic mini‐pumps, the mice were anesthetized with isoflurane (Mylan Inc.) using an animal anesthesia machine (DS Pharma Biomedical Co. Ltd.; induction: concentration = 4%, flow rate = 2.8 L/min; maintenance: concentration = 2%, flow rate = 2.8 L/min). Anesthetized mice were placed on a heated pad maintained at 35°C. At the end of the experiment, the mice were sacrificed via carotid artery bleeding under inhalation anesthesia with isoflurane (Mylan Inc.).

### 2.6 Cell culture and ultrasound irradiation of HUVECs

Two batches of human umbilical vein endothelial cells (HUVECs) were purchased from LONZA Ltd. (Basel, Switzerland; Cat. #C2519), corresponding to Lonza's Clonetics™ HUVECs derived from multiple donors. Cells were cultured in EGM‐2MV complete medium (LONZA, Cat. #CC‐3202) and expanded up to passage 3. All experiments were performed using cells at passage 5. After 24 h of culture in a 6‐well plate (3.3 × 10^5^ cells/well; Corning Inc., Corning, NY, USA; Cat. #3506), the HUVECs were serum‐starved for 16 h. For the inhibition studies, cells were pre‐treated with an FAK inhibitor (3 μM PF‐573228; Sigma Aldrich; Cat. #PZ0117), which selectively inhibits FAK, or a PLC inhibitor (1 μM U 73122; Cayman Chemical, Ann Arbor, MI, USA; Cat. #70740), a widely used PLC inhibitor, for 3 h prior to PUS stimulation. These concentrations and treatment durations were selected based on previous literature (Cabrita et al., 2011; Nishitani et al., 2011) and preliminary dose–response experiments to ensure effective inhibition without inducing cytotoxicity. The culture plate was then placed gently onto solid ultrasound gel (Toshiba Medical Supply Co. Ltd., Tokyo, Japan) for PUS irradiation. Transducers were connected to the main controller (Nippon Sigmax). Ultrasound exposure conditions were as follows: intensity of 30 mW/cm^2^, transducer effective area of 6.5 cm^2^, pulse frequency of 2.0 MHz with a 200‐μs pulse duration (20% duty cycle), 1‐kHz repeat rate, and exposure time of 20 min. HUVECs were collected 0, 5, 10, and 20 min after ultrasound exposure.

### 2.7 Tube formation assay

Growth factor reduced/phenol red free matrigel (BD Bioscience; Cat. #356231) was placed in the wells of μ‐slide angiogenesis (ibidi GmbH, Planegg, Germany; Cat. #81506). HUVECs were seeded (2.5 × 10^3^ cells/well) onto the matrigel. One hour after seeding, the cells were exposed to ultrasound for 20 min. For the blocking antibody inhibition assay, a β_1_ integrin‐blocking antibody (1.0 μg/mL; BV7, Abcam plc, Cambridge, UK; Cat. #ab7168) was added to the media. As a control, an isotype‐matched mouse IgG1 antibody (1.0 μg/mL; Abcam plc; Cat. #ab170190) was used under the same conditions to account for non‐specific antibody effects. The cells were observed after 4 h from PUS irradiation. Quantification was done with ImageJ (Rasband, W.S., ImageJ, National Institutes of Health, Bethesda, Maryland, USA, http://imagej.nih.gov/ij/, 1997–2014). Details of antibodies used for tube formation assay are provided in Table S1.

### 2.8 PathScan RTK signaling

A PathScan RTK Signaling Antibody Array Kit (Cell Signaling Technology, Inc., Danvers, MA, USA; Cat. #7982) was used according to the manufacturer's instructions. Briefly, HUVECs were serum‐starved for 16 h and then irradiated with PUS for 5, 10, or 20 min. Cells exposed for 5 or 10 min were collected immediately. Cells that were exposed for 20 min were incubated for an additional 0, 5, 10, or 20 min. The cells were lysed in Radio‐Immunoprecipitation Assay (RIPA) buffer (Cell signaling Technology), and the cell lysates were analyzed with the antibody array. Dots of phosphorylated target proteins were detected with Chemidoc (Bio‐Rad Laboratories, Inc., Hercules, CA, USA). Densitometric analysis was performed with Image Lab software (Bio‐Rad).

### 2.9 Western blotting

HUVECs were washed with ice‐cold PBS, collected with a cell scraper, and lysed with RIPA buffer supplemented with protease inhibitor cocktail (1 tablet/50 mL; Roche Diagnostics GmbH, Mannheim, Germany; Cat. #11697498001) and phosphatase inhibitor cocktail (1 tablet/10 mL; Roche Diagnostics GmbH, Cat. #04906837001) and boiled for 5 min under reducing conditions. The samples were resolved with 10% sodium dodecyl sulfate‐polyacrylamide gel electrophoresis and transferred to a PVDF membrane. The membranes were blocked with 5% BSA in TBS‐T (20 mM Tris–HCl, 300 mM NaCl, 0.05% Tween 20, pH 7.5) at room temperature for 1 h and incubated at 4°C overnight with primary antibodies. The list of employed antibodies and their concentrations is given in Table S1. The membranes were incubated with the corresponding horseradish peroxidase‐conjugated secondary antibodies. The immunoreactive bands were visualized with an ECL western blotting system (GE Healthcare UK Ltd., Buckinghamshire, UK; Cat. #RPN2106) and detected with Chemidoc (Bio‐Rad). Densitometric analysis was performed with Image Lab software (Bio‐Rad). The level of phosphorylated ERK1/2 was normalized to that of total ERK1/2. Protein molecular weight was estimated using three different standards depending on the figure panels. For Figure 2a,e, as well as Figure 3a,e, Precision Plus Protein™ WesternC™ Standards (Bio‐Rad; Cat. #1610376) were used. For Figures 2c, 3g, and 4b, Precision Plus Protein™ Kaleidoscope Standards (Bio‐Rad; Cat. #1610375), a pre‐stained visible marker, were used for direct visualization. For Figure 3c, MagicMark™ XP Western Protein Standard (Thermo Fisher Scientific; Cat. #LC5602) was used. Molecular weight positions of marker ladder proteins were used to estimate band sizes and are indicated on the left side of each blot.

### 2.10 Immuno‐fluorescent assay

Glass‐bottom dish was coated with fibronectin (5 μg/mL; Sigma‐Aldrich; Cat. #F0895; derived from human plasma). Then HUVECs were seeded (5.0 × 10^3^ cells) onto the glass bottom dish (Matsunami Glass Ind., Ltd., Osaka, Japan; Cat. #D11130H). After PUS irradiation, cells were fixed with 4% formaldehyde, permeabilized with triton‐X100 and blocked with 1% BSA in PBS. The cells were stained with anti‐active integrin β_1_ antibody (Clone 12G10; Cell Signaling Technology, Cat. #53550) and Alexa488 labeled anti‐mouse IgG antibody (Thermo Fisher Scientific Inc., Waltham, MA, USA). Actin filament was stained with phalloidin (Acti‐stain 555, Cytoskelton, Inc., Denver CO, USA; Cat. #PHDH1). After washing with PBS, cells were mounted in VECTASHIELD with DAPI (Vector Laboratories, Inc., Burlingame, CA, USA; Cat. #H‐1200) and examined by fluorescence microscopy. Details of antibodies used for Immuno‐Fluorescent assay are provided in Table S1.

### 2.11 Statistical analysis

Data are expressed as means ± standard deviation (SD). Multiple in vivo comparisons were performed using the Friedman test followed by Holm's test. The significance of differences between the control and PUS groups was determined with the Student's *t*‐test. The significance of differences between the control and PUS groups in vitro was determined with the Kruskal‐Wallis test followed by Steel's multiple comparison tests. Data were processed using the Ekuseru‐Toukei 2012 software (Social Survey Research Information Co. Ltd., Tokyo, Japan). Bar charts were generated using JASP statistical software (Version 0.95.3; JASP Team, Amsterdam, The Netherlands).

## 3 RESULTS

### 3.1 PUS‐induced blood flow recovery and angiogenesis in a mouse hindlimb ischemia model

**FIGURE 1:**
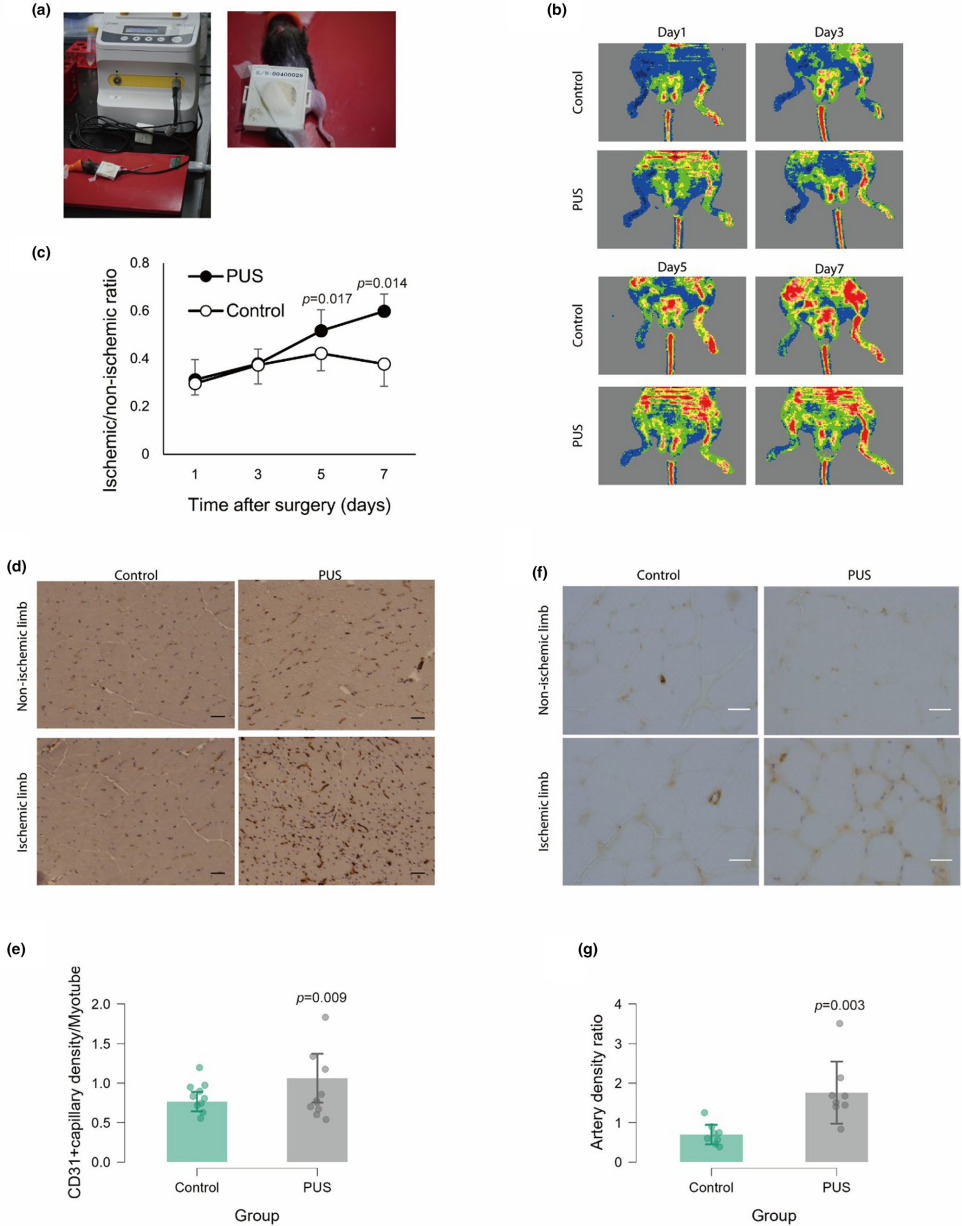
Effect of pulsed ultrasound (PUS) on blood flow recovery in hindlimb ischemic model mice. Ischemia was surgically induced in the right hindlimbs. Surgery was not performed on the left hindlimbs (as reference of blood flow). (a) In the PUS group, the right hindlimbs were irradiated with PUS for 20 min/day for 14 days. (b) Representative laser Doppler images after surgery. A color scale illustrates blood flow variations from minimal (dark blue) to maximal (red) values. (c) Quantitative analysis of the ischemic/non‐ischemic limb laser Doppler imaging (LDI) ratio (*n* = 10 per group). The data represent means ± standard deviation. (d) The ischemic and nonischemic adductor muscles were sectioned and immunostained with anti‐CD31 antibody. Original magnification, ×200. (e) Quantitative analysis of the ischemic/nonischemic limb CD31‐positive capillary density ratio. (f) Representative image of immunohistochemistry analysis for smooth muscle Actin in ischemic and non‐ischemic femur muscles. Original magnification, ×400. (g) Quantitative analysis of the ischemic/nonischemic limb alpha smooth muscle Actin‐positive artery density ratio. The data represent means ± standard deviation. For (d, f) scale bar = 100 μm. Static analysis was performed using the Friedman test followed by Holm's test (c) and the Student's *t*‐test (e, g).

We constructed a device for PUS therapy (Figure S1). To verify the effects of PUS on angiogenesis in vivo, we created a mouse hindlimb ischemia model via femoral artery ligation and applied PUS irradiation for 20 min/day for 1 week. One transducer element per mouse was applied to irradiate PUS (Figure 1a). The blood flow rate declined at the ischemic hindlimb on the day after surgery (day 1). Compared with the control (no PUS treatment) group mice, mice in the PUS group had a significantly increased blood flow rate on days 5 and 7 (day 5, PUS: 0.52 ± 0.03, control: 0.42 ± 0.07; day 7, PUS: 0.60 ± 0.07, control: 0.38 ± 0.09) (Figure 1b,c). To evaluate the effects of PUS on the recovery of blood flow rate, we verified the capillary densities of hindlimb muscle sections with immunohistochemical staining against CD31 and α smooth muscle actin (αSMA). As shown in Figure 1d,e, the capillary density rate was higher in the PUS group than in the control group. Moreover, compared with the control group, the PUS group showed significant regeneration of αSMA‐positive arteries (Figure 1f,g). These findings suggested that PUS stimulation induced microvessel proliferation and facilitated blood flow recovery after ischemic surgery. These results collectively demonstrate that PUS stimulation significantly enhances angiogenic responses and promotes functional recovery of blood perfusion in ischemic hindlimb tissue.

### 3.2 PUS‐activated ERK1/2 via FAK in HUVECs

**FIGURE 2:**
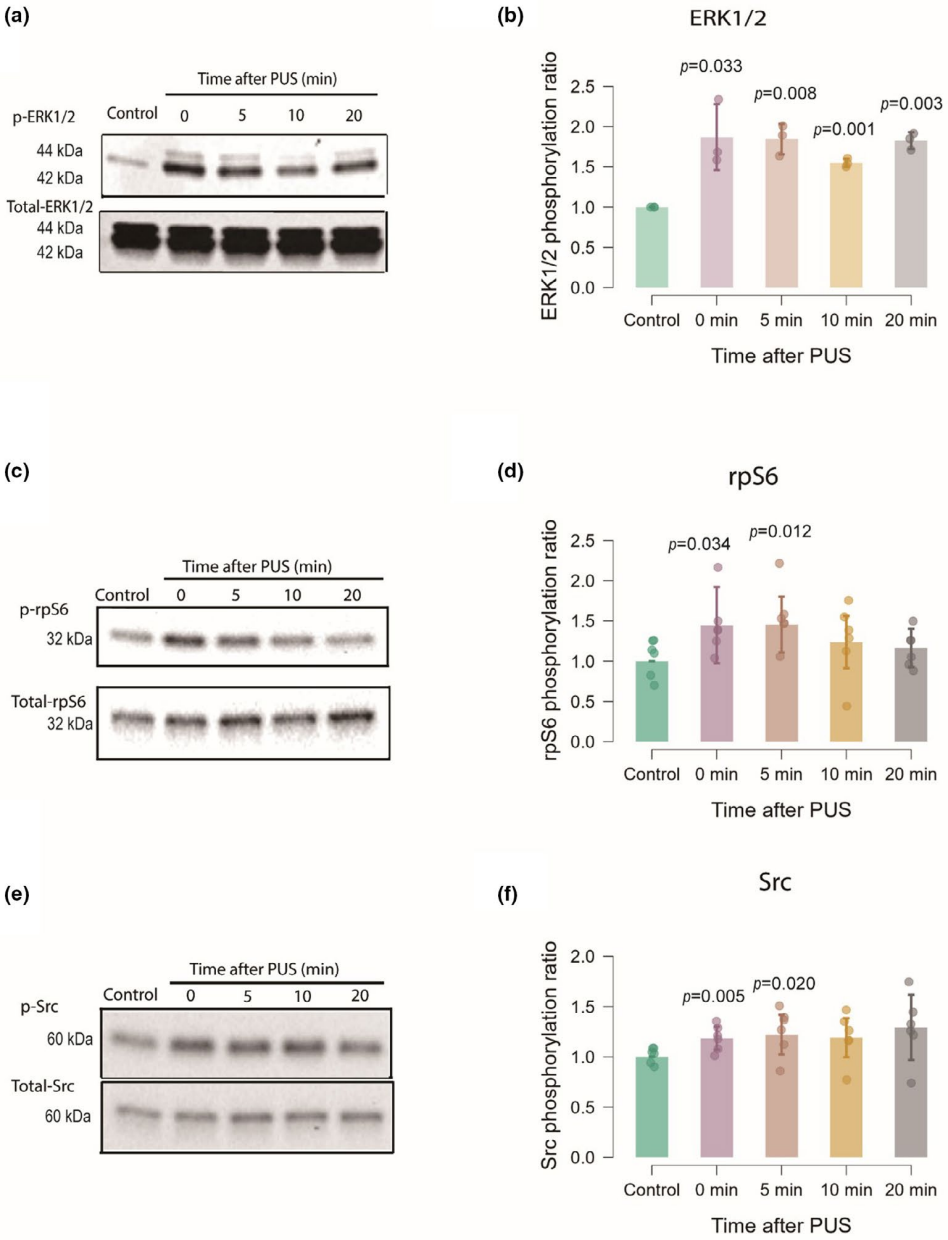
PUS induces phosphorylation of ERK1/2, ribosomal protein S6 (rpS6), and Src. HUVECs (3.3 × 10^5^ cells/well) were exposed to PUS for 20 min and incubated for 0‐, 5‐, 10‐, or 20‐min (*n* = 3). Whole‐cell lysates were blotted with phospho‐specific antibody against ERK1/2 (a, b), rpS6 (c, d), and Src (e, f). The intensities of immunoreactive bands were quantified by scanning. See Figure S3 for full blot images including marker positions. The data represent means ± standard deviation. Static analysis was performed using the Kruskal‐Wallis test followed by Steel's multiple comparison tests.

To elucidate the signal transduction mechanism initiated by PUS stimulation in HUVECs, we evaluated phosphorylation profiles with antibody array analysis. HUVECs were exposed to PUS for 5, 10, or 20 min. Cells exposed to PUS for 20 min were harvested at 0, 5, 10, and 20 min after PUS stimulation. Cell lysates were obtained for antibody array analysis. Positive dots were detected as ERK1/2, ribosomal protein S6 (rpS6), and Src (Figure S2). The phosphorylation ratio of c‐Abl was not meaningfully increased with or without PUS, whereas the phosphorylation of rpS6, Src, and ERK1/2 was markedly increased. These phosphorylation ratios increased in a PUS exposure time‐dependent manner and gradually decreased after the cessation of PUS treatment. Figure 2 shows the phosphorylation of ERK1/2, rpS6, and Src confirmed with western blotting analysis. These results implied that PUS stimulation activated ERK1/2, rpS6, and Src.

**FIGURE 3:**
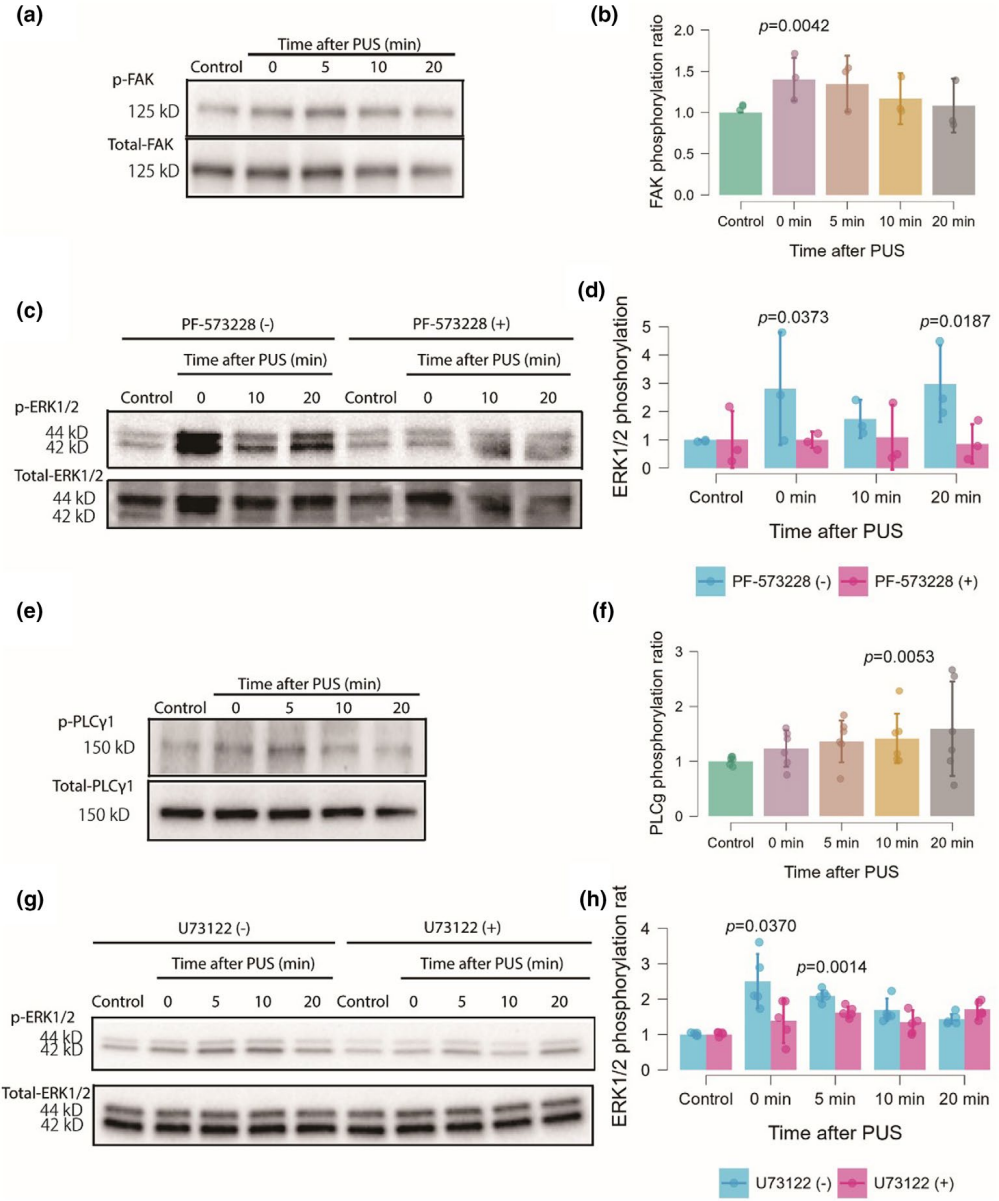
PUS induces FAK and PLCγ1 in HUVECs. HUVECs (3.3 × 10^5^ cells/well) were exposed to PUS for 20 min and incubated for 0‐, 5‐, 10‐, or 20‐min. Whole‐cell lysates were blotted with phospho‐specific antibody against FAK (a, b) or PLCγ1 (e, f). The intensities of immunoreactive bands were quantified by scanning. HUVECs were pre‐treated with (c, d) the FAK inhibitor PF‐573288 (3 μM, *n* = 3) or (g, h) the PLC inhibitor U73122 (1 μM, *n* = 4). After pre‐treatment, the cells were exposed to PUS for 20 min and incubated for 0, 5, 10, or 20 min. Phosphorylated ERK1/2 was detected with western blotting analysis. See Figure S3 for full blot images including marker positions. Data represent means ± standard deviation. Static analysis was performed using the Kruskal‐Wallis test followed by Steel's multiple comparison tests.

Focal adhesion components such as integrin and Src reportedly induce phosphorylation of ERK1/2 with PUS (Zhou et al., 2004). Src is phosphorylated by FAK, which is activated by integrin. In addition, FAK upregulates the phosphorylation of PLCγ1 (Sieg et al., 1999; Tvorogov et al., 2005; Zhang et al., 1999). Because Src is activated with PUS, we tested whether PUS‐induced ERK1/2 phosphorylation was stimulated via focal adhesion components (Figure 3). PUS augmented the phosphorylation of FAK and PLCγ1. Moreover, to determine whether FAK and PLCγ1 are associated with ERK1/2 activation, we pre‐treated PUS irradiated cells with the FAK inhibitor PF‐573228 (Slack‐Davis et al., 2007) or the PLC inhibitor U73122. We found that ERK1/2 phosphorylation by PUS was suppressed by either PF‐573228 or U73122 treatment (Figure 3c,d,g,h). These findings suggested that the stimulation of ERK1/2 by PUS was mediated by focal adhesion components—namely FAK, Src, and PLCγ1. These findings collectively indicate that PUS stimulation activates ERK1/2 signaling in HUVECs through a focal adhesion‐dependent pathway involving FAK, Src, and PLCγ1. This mechanistic insight suggests that PUS may modulate endothelial cell function via integrin‐mediated signal transduction.

### 3.3 PUS‐induced angiogenesis attenuated by integrin blocking

**FIGURE 4:**
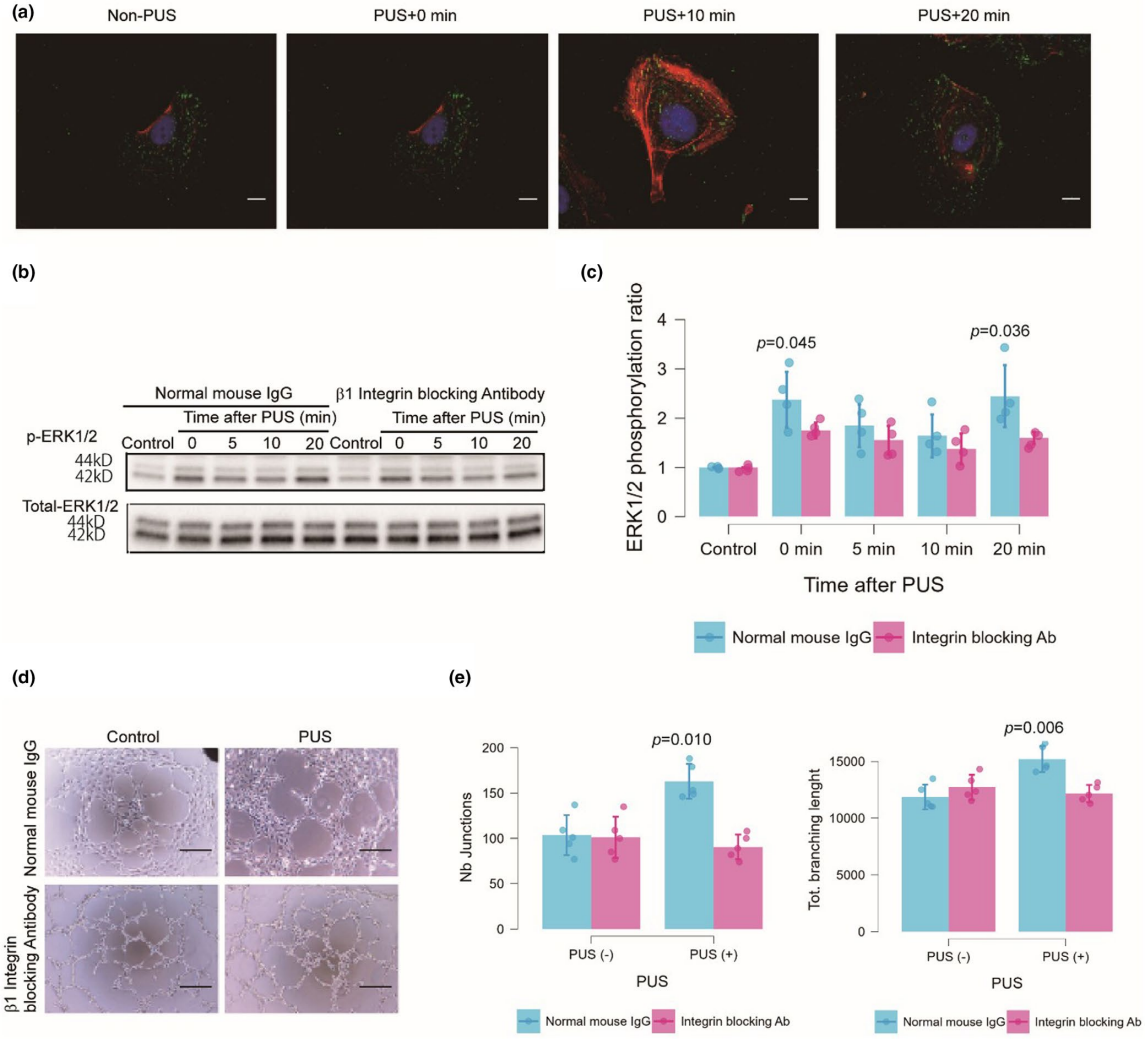
Pulsed ultrasound (PUS) treatment induces ERK1/2 activation for tube formation through the integrin signaling pathway. Immunofluorescent assay for active β_1_ integrin and filamentous actin (F‐actin) in human umbilical vein endothelial cells (HUVECs) (a). HUVECs were treated with PUS for 20 min, and β_1_ integrin and F‐actin were investigated by immunofluorescent staining. Anti‐integrin β_1_ antibody was detected with secondary antibody conjugated with Alexa Fluor 488 (green), while F‐actin was stained with Acti‐stain™ 555 phalloidin (red). Immunoblot assay for phosphorylation of ERK1/2 after PUS irradiation on HUVECs treated with anti‐integrin β_1_ neutralizing antibody (b). HUVECs (3.3 × 10^5^ cells/well) were pre‐incubated with anti‐β_1_ integrin antibody (10 μg/mL) or normal mouse IgG (10 μg/mL) for 3 h. PUS irradiation was applied for 20 min. The cells were collected 0, 5, 10, or 20 min after PUS stimulation. Phosphorylation ERK1/2 was quantified with scanning densitometry of immunoreactive bands (c). See Figure S3 for full blot images including marker positions. *n* = 4. For the tube formation assay, HUVECs were incubated with anti‐β_1_ integrin antibody (10 μg/mL) or normal mouse IgG (10 μg/mL). The cells were exposed to PUS for 20 min. Four hours after the PUS irradiation, tube formation was observed (d). Junction numbers were measured in each well using ImageJ (e). The data are shown as means ± standard deviation. *n* = 3. For (a) scale bar = 20 μm, for (d) scale bar = 100 μm. Static analysis was performed using the Kruskal–Wallis test followed by Steel's multiple comparison tests.

Using the results of PUS‐induced signal transduction analysis (see Figures 2 and 3), we hypothesized that integrin plays a critical role in the response of endothelial cells to PUS because PUS stimulates β_1_ integrin (Huttenlocher & Horwitz, 2011; Mahoney et al., 2009; Tang et al., 2006; Watabe et al., 2011). To monitor HUVEC expression of integrin β_1_, we performed an immunofluorescent staining assay. The HUVECs were stained with an anti‐active integrin β_1_ monoclonal antibody and phalloidin for actin polymerization (Figure 4a). Integrin β_1_ and F‐actin levels drastically increased at the end of the PUS irradiation (PUS +0 min). However, 20 min after the PUS exposure ended, these activations were reduced. Moreover, we carried out a PUS irradiation assay with integrin‐inhibited HUVECs using a β_1_ integrin‐blocking antibody (Figure 4b,c). The PUS‐induced phosphorylation of ERK1/2 was markedly attenuated in antibody‐treated HUVECs. Tube formation assays were also performed using a β_1_ integrin‐blocking antibody (Figure 4d,e). Whereas PUS increased tube formation in HUVECs (Figure 2), the tube formation enhancement induced by PUS was completely abrogated by the anti‐β_1_ integrin‐blocking antibody. These data suggested that β_1_ integrin mediated PUS‐induced angiogenesis in vitro. Taken together, these results demonstrate that β1 integrin plays a pivotal role in mediating the cellular response to PUS stimulation in endothelial cells. The activation of β1 integrin is essential for downstream ERK1/2 phosphorylation and angiogenic behavior, including tube formation, suggesting that integrin‐dependent mechanotransduction is a key mechanism underlying PUS‐induced angiogenesis in vitro.

### 3.4 PUS‐induced blood flow recovery inhibited by administration of anti‐β_1_ integrin‐blocking antibody

**FIGURE 5:**
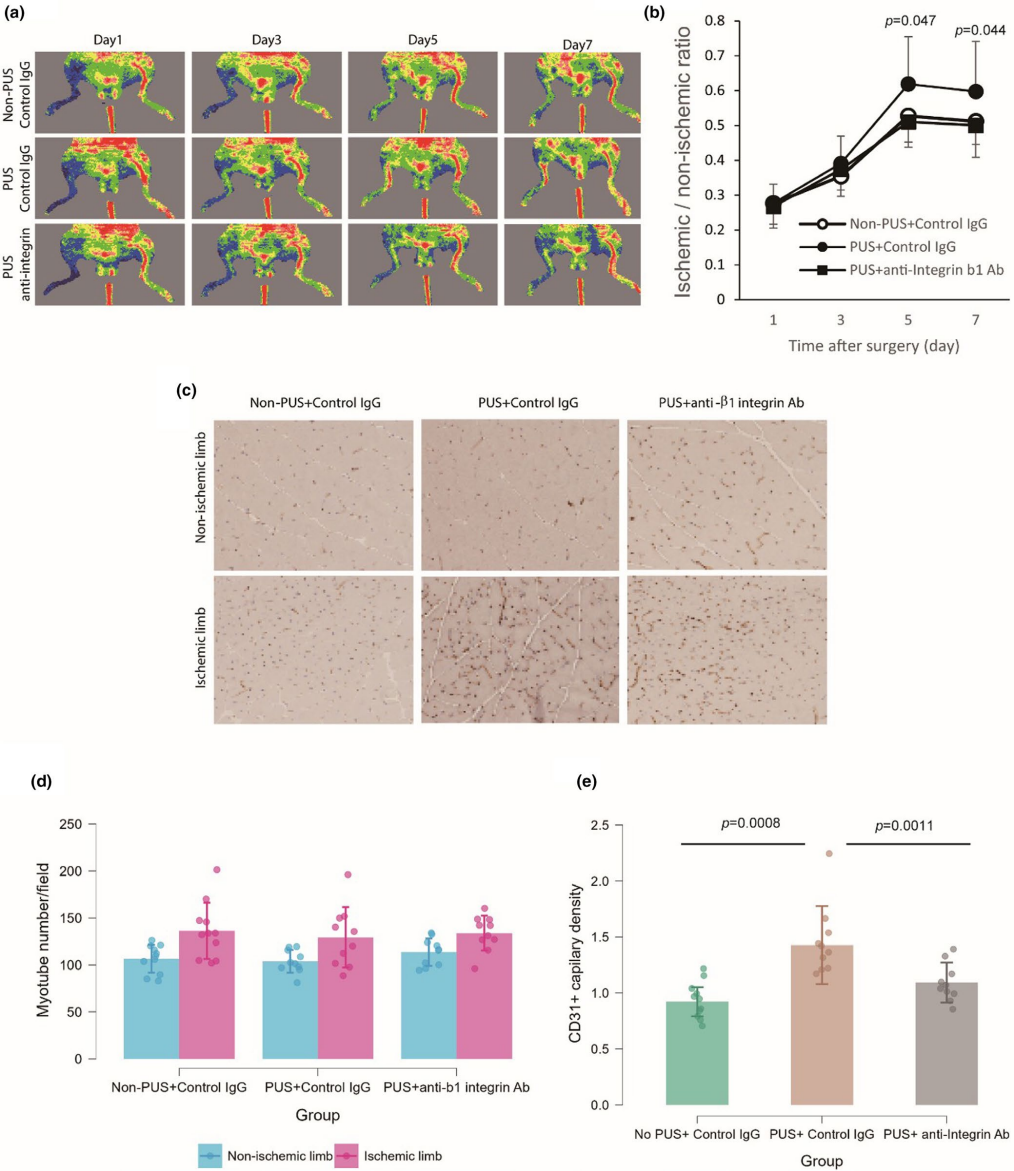
Effects of Pulsed Ultrasound (PUS) on Angiogenesis and Integrin Pathway in Hindlimb Ischemic Mice. Hindlimb ischemic mice were divided randomly by bodyweight into three groups (*n* = 10 per group): PUS with control IgG, PUS with anti‐β_1_ integrin antibody, and non‐PUS with control IgG. Mini‐osmotic pumps containing either anti‐β_1_ integrin antibody (1 mg/mL, 100 μL) or isotype control IgG (1 mg/mL, 100 μL) were implanted subcutaneously in the backs of all mice. The day after surgery, mice were exposed to PUS once a day for 20 min. Blood flow was measured with LDI every 2 or 3 days (a, b). Data represent means ± standard deviation. At the end of the study period, both left and right femur muscles were dissected and immunostained for CD31 to assess vascularity (c). Original magnification, ×200. Myotube number per field of five different microscopic fields (d). Ischemic/nonischemic capillary density was calculated with the ratio of CD31‐positive capillary number to number in five different microscopic fields (e). The data represent means ± standard deviation. *n* = 10. For (c) scale bar = 100 μm. Static analysis was performed using the Friedman test followed by Holm's test (b), the Student's *t*‐test (d) and the Holm's test (e).

To evaluate whether β_1_ integrin is associated with PUS‐induced blood flow recovery in vivo, we continuously administered a blocking antibody against β_1_ integrin to ischemic model mice (Figure 5a,b). The blood flow rate recovered remarkably in the PUS group on days 5 and 7 (day 5, Non‐PUS+Control IgG: 0.53 ± 0.07, PUS+Control IgG: 0.62 ± 0.14; day 7, Non‐PUS+Control IgG: 0.51 ± 0.07, PUS+Control IgG: 0.60 ± 0.14). However, no significant blood flow restoration was observed in the group administered the β_1_ integrin‐blocking antibody (PUS+anti‐Integrin b1 Ab, day 5: 0.51 ± 0.07, day 7: 0.50 ± 0.09). Furthermore, we performed an immunostaining assay to measure capillary density in the femur muscles of ischemic mice (Figure 5c–e). There was no significant difference in myofiber numbers among the control, PUS with control IgG, and PUS with anti‐integrin β_1_ antibody group (Figure 5d). Meanwhile, the capillary density in the PUS with control IgG group mice (1.43 ± 0.34) was 1.5‐fold higher than that of non‐PUS group mice (0.92 ± 0.13). Nevertheless, no significant difference was found between the PUS with anti‐β_1_ integrin group (1.11 ± 0.18) and the non‐PUS group (Figure 5e). These data suggested that blood flow recovery provided by PUS treatment was prevented by the anti‐β_1_ integrin antibody. These findings indicate that β1 integrin is essential for the therapeutic effects of PUS in vivo. Blocking β1 integrin abrogated PUS‐induced blood flow recovery and capillary formation in ischemic muscle, suggesting that integrin‐mediated mechanotransduction is a critical pathway for PUS‐enhanced angiogenesis and tissue perfusion.

## 4 DISCUSSION

Cells transfer physiological force such as compression, tension, or shear stress to a biochemical or genetic response via mechanosensors (Orr et al., 2006). Primary cilia, transient receptor potential vanilloid 4, and integrins are mechanosensitive proteins that are stimulated by ultrasound (Filosa et al., 2013; Malone et al., 2007; Schwartz & DeSimone, 2008). Mechanical stress caused by blood flow or blood pressure stimulates vascular endothelial cells and induces cytoskeletal remodeling via Rho‐ROCK, MAPK, and Wnt/β‐catenin signaling (Civelekoglu‐Scholey et al., 2005; Dejana, 2010; Ruwhof & van der Laarse, 2000). Mechanical stimulation with PUS enhanced integrin conformational activation and triggered actin polymerization (Figure 4a). Integrin and actin filament induce Ca^2+^ influx as mechanosensitive channels in HUVECs (Hayakawa et al., 2008; Kawakami et al., 2001). The calcium influx triggered by mechanosensitive structures such as integrins and actin filaments plays a pivotal role in converting mechanical stimuli into biochemical signals. In vascular endothelial cells, this calcium signaling cascade contributes to cytoskeletal remodeling and angiogenic responses, thereby serving as a key mediator of mechanotransduction. It was previously reported that there were more blood vessels in ultrasound‐treated wounds than in the control wounds within 5 days after injury in rat (Young & Dyson, 1990) and that ultrasound induced the expressions of angiogenic factors in human fibroblasts, osteoblasts, and monocytes (Doan et al., 1999). Therefore, we tested PUS on ischemic model. We first investigated whether PUS exposure induced blood flow recovery in hindlimb ischemic mice. Then, we examined whether PUS stimulates ERK1/2 via the integrin/FAK pathway in HUVECs. The integrin signal pathway mediates mechanotransduction (Harburger & Calderwood, 2009; Shyy & Chien, 2002).

Anti‐integrin antibody markedly suppressed blood flow recovery and capillary density in the ischemic mouse model. Because the hindlimb ischemia mouse model is an acute ischemia model, it might be inadequate for the study of PAD. However, it is a well‐established model that has been used in several studies for the development of PAD therapy (Hellingman et al., 2010; Kim et al., 2006; Limbourg et al., 2009). Hence, we used this animal model to obtain data with which to evaluate the contribution of PUS to the treatment of PAD. It is reported that some risk factors such as hypertension or diabetes lead to courses of PAD (Higashi et al., 2016). To assess these symptoms, because most of the model mice for these symptom origins are C57BL/6 (Arruda et al., 2005), we need to validate whether PUS affects the ischemic hindlimb of C57BL/6 or not. In the future, we will perform the effects of PUS on various conditions including aging or diabetic symptoms.

Animals were anesthetized with isoflurane for their safety because of daily use. Actually, isoflurane could influence tissue perfusion, so the control group was also anesthetized with isoflurane. Because the anesthetic depth of injectable anesthesia will vary during measurements, hemodynamics such as blood pressure will not be stable during LDI measurement (Buitrago et al., 2008). In contrast, isoflurane will provide stable cardiovascular physiology (Constantinides et al., 2011; Greco et al., 2013).

Meanwhile, general anesthesia suppresses the central nervous system and decreases body temperature. Therefore, during anesthesia, the mice were recommended to be placed on a heating pad to keep body temperature constant, because the anesthetic agent could induce hypothermic (Caro et al., 2013).

It has been reported that shock wave improved hindlimb ischemia (Holfeld et al., 2014; Tepekoylu et al., 2013). LIPUS affected myocardial ischemia (Hanawa et al., 2014). Therefore, mechanical stress could improve blood flow. In this report, we apply PUS as a mechanical stress to angiogenesis.

Although the ischemic muscle in the PUS group shows more capillaries than in the other groups, myofiber numbers were not significantly different. The muscle fibers of ischemic muscle are much smaller and the nuclei are centrally located in the myofibers (Figure 5c). The muscle seems to be regenerating due to ischemic injury. This is a hallmark of regenerating muscle (Folker & Baylies, 2013).

The results of this study showed that PUS stimulated phosphorylation of ERK1/2 in human endothelial cells (see Figures 2a,b, 3c,d,g,h, and 4b,c). ERK1/2 regulation is involved in many processes, including cell adhesion, cell cycle progression, cell migration, cell survival, and differentiation (Roskoski Jr., 2012). Therefore, tube formation might be enhanced in HUVECs stimulated with PUS in addition to secondary effects in PUS‐activated fibroblasts. We evaluated that FAK associated with ERK activation by PUS with FAK inhibitor (see Figure 3c,d). FAK plays a pivotal role in the integrin signaling pathway. Src binds to phosphorylated FAK. ERK is well‐known to be one of the targets of FAK‐Src (Saleem et al., 2009; Schlaepfer et al., 1999). Our results indicated that PUS enhanced the functions of FAK and Src, which correlate with integrin activity (Mitra et al., 2005). We showed that PUS stimulation induces PLCγ1 activation. Inhibition of PLCγ1 prevented PUS‐induced ERK1/2 activation (see Figure 3e–h). These data suggested that PLCγ1 had a role in mediating the positive effects of PUS on ERK1/2 activation. PLCγ1 is a regulator of ERK1/2 that influences cell proliferation (Olsson et al., 2006). It is also essential for early events in integrin signaling, which is required for cell motility (Jones et al., 2005). Thus, PLCγ1 might play a key role in the PUS‐induced proliferation and migration of endothelial cells. We showed that PUS stimulation induced rpS6 phosphorylation. ERK1/2 promotes rpS6 phosphorylation through ribosomal S6 kinase and stimulates translation (Roux et al., 2007). This integrin signaling pathway should enhance tube formation by PUS in HUVECs (see Figure 4d,e) (Arnaoutova & Kleinman, 2010; Donovan et al., 2001).

Recent study reported that ultrasound (1 MHz, 300 mW/cm^2^, 9 min per day) improved perfusion in ischemic mice and enhanced expression of angiogenic factors such as eNOS, VEGF, and HIF‐1α (Huang et al., 2014). Ultrasound at 300 mW/cm^2^ could increase temperature; meanwhile, PUS at 30 mW/cm^2^ should not significantly increase temperature (Toyama et al., 2012). We treated mice with PUS as 2 MHz, 30 mW/cm^2^, and 20 min per day. In a simulation model to predict the thermal effects of PUS at 30 mW/cm^2^ on human skeletal muscle tissue, the PUS treatment used in the present study increased core muscle temperature by a maximum of 0.006°C. Specifically, skin temperature did not significantly change in PUS‐exposed legs (36.1 ± 0.3 to 36.2 ± 0.4°C, *p* = 0.79) compared with non‐exposed legs (36.3 ± 0.4 to 36.3 ± 0.4°C, *p* = 0.88). These findings provide new insights into the mechanism of PUS‐induced endothelial vessel formation and suggest that PUS has therapeutic benefits in patients with PAD. In addition, another noninvasive technique, extracorporeal shock wave therapy, has been used to treat PAD (Cayton et al., 2016; Ciccone et al., 2012). Significant improvements in pain‐free and maximum walking distances were observed. This finding suggests that ESWT promotes angiogenesis through physical stimulation.

**FIGURE 6:**
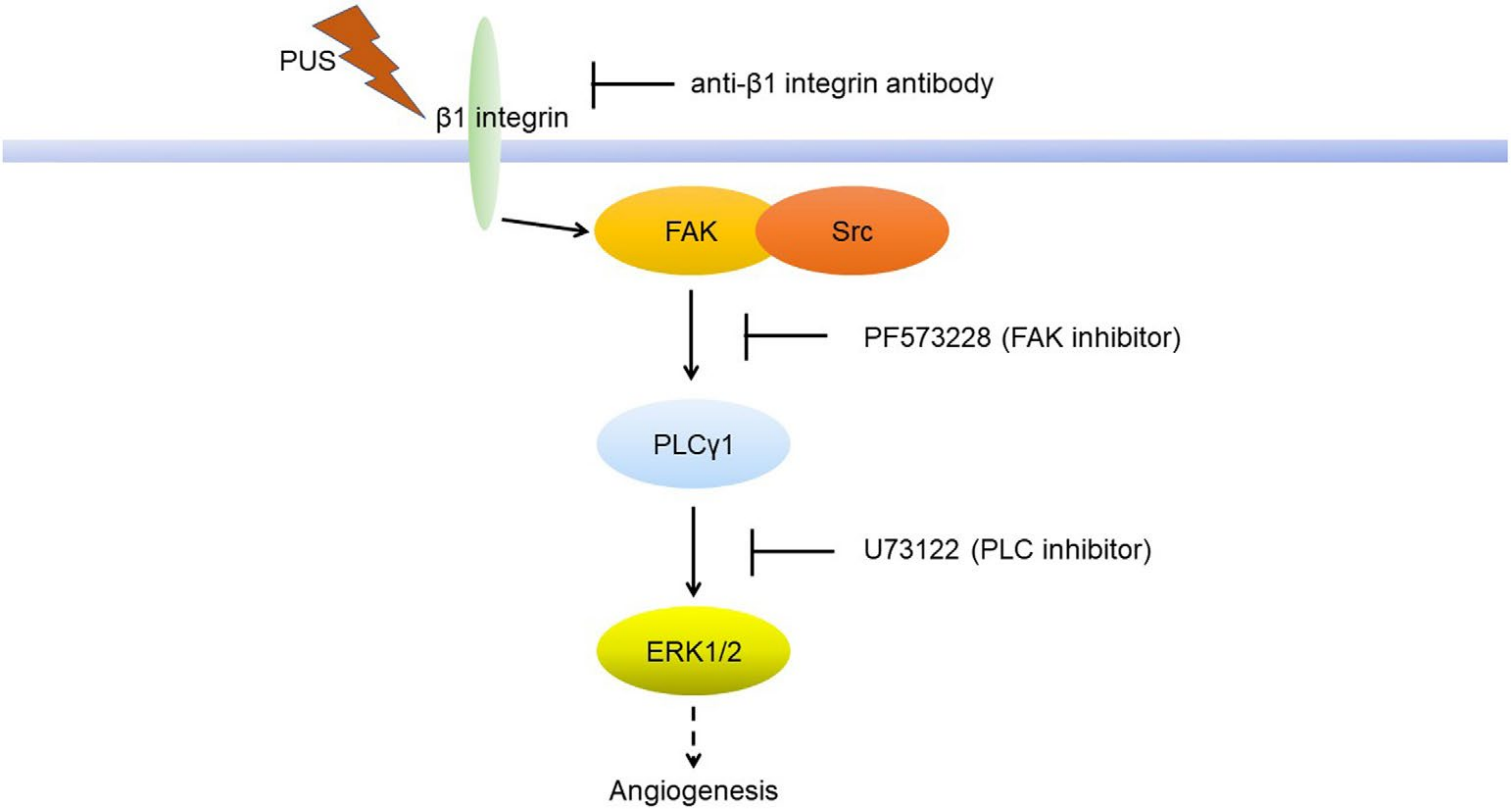
Proposed signaling pathway activated by pulsed ultrasound (PUS) in endothelial cells. Schematic diagram illustrating the signaling cascade investigated in this study. PUS stimulation activates β1 integrin, which subsequently triggers downstream signaling involving FAK, Src, and PLCγ1, leading to ERK1/2 phosphorylation. This pathway contributes to endothelial tube formation and angiogenic responses.

In this study, we elucidated that the integrin/FAK signaling pathway contributes to angiogenic activity with PUS (see Figures 3, 4, 5). To summarize the proposed mechanism, a schematic diagram illustrating the signaling cascade involving β1 integrin, FAK, Src, PLCγ1, and ERK1/2 has been included as Figure 6. The blocking antibody against β_1_ integrin suppressed PUS‐induced ERK1/2 activation and attenuated PUS‐induced vessel formation. These findings indicated that β_1_ integrin may be a critical regulator of PUS signaling in the activation of endothelial cells. β_1_ integrin and β_3_ integrin are expressed on endothelial cells including HUVEC (Baranska Hj et al., 2005; Conforti et al., 1992; Dejana, 2004; Dejana et al., 1993; Urbich et al., 2000). β1 integrin induces phosphorylation of FAK and ERK1/2 by shear stress (Urbich et al., 2002). Hence, combination therapy with PUS and integrin ligands may have a synergistic effect on endothelial activation and angiogenesis. Moreover, integrin may represent a novel target for therapeutic angiogenesis.

This study has a limitation in that only a single ultrasound intensity and time course were evaluated. While this approach allowed us to establish a baseline biological response under a clinically relevant condition, it does not capture the potential dose‐dependent effects of LIPUS. Future studies should investigate a range of intensities and treatment durations to better understand the optimal parameters for therapeutic efficacy.

PUS irradiation is a noninvasive strategy. Indeed, no severe adverse effects were observed after PUS irradiation in the present study. However, prospective randomized controlled clinical trials are required to evaluate adverse effects, including cardiovascular outcomes and the onset of malignancy. Therefore, it may be a candidate for a new therapy to improve ischemic symptoms.

In conclusion, this study demonstrates that PUS promotes angiogenesis through integrin‐mediated mechanotransduction in endothelial cells. PUS activates β1 integrin, leading to downstream phosphorylation of FAK, Src, PLCγ1, and ERK1/2, which collectively enhance endothelial cell functions such as tube formation and capillary regeneration. In vivo, PUS significantly improved blood flow and capillary density in a mouse hindlimb ischemia model, effects that were abrogated by β1 integrin blockade. These findings suggest that PUS is a promising non‐invasive therapeutic strategy for ischemic vascular diseases such as PAD, and that integrin signaling may serve as a key target for enhancing angiogenic responses. Further studies are warranted to evaluate the safety and efficacy of PUS in chronic ischemic conditions and in clinical settings.

## AUTHOR CONTRIBUTIONS

YH had full access to all the data in the study and takes responsibility for the integrity of the data and the accuracy of the data analysis. Study concept and design: YH. Drafting of the manuscript: YK and YH. Designed animal studies and cell studies: YK and YH. Acquisition and analysis of animal studies data: YK and FM. Acquisition and analysis of histological studies data: YK and KK. Acquisition and analysis of cell studies data: YK. Obtained funding: YH. Study supervision: YH. All authors read and approved the final article.

## FUNDING INFORMATION

This work was supported in part by a Grant‐in‐Aid for Scientific Research from the Ministry of Education, Science and Culture of Japan [1859081500, 21590898].

## CONFLICT OF INTEREST STATEMENT

Yoshitsugu Kojima is an employee of Nippon Sigmax Co. Ltd. Fumitaka Mizuki is an ex‐employee of Nippon Sigmax Co. Ltd. Ki‐ichiro Kawano and Yukihito Higashi declare no potential conflict of interest.

## Supporting information


Figures S1–S3.

Table S1.

## Data Availability

The data are available from the corresponding author upon reasonable request.
