# Supplementary material for: Low‐intensity ultrasound induces angiogenesis by activating endothelial integrin signaling in male mice
Source: Physiol Rep. 2026 Jun 26;14(12):e70718. doi: 10.14814/phy2.70718 (PMC13305677; doi:10.14814/phy2.70718)

# **SUPPLEMENTAL MATERIAL**


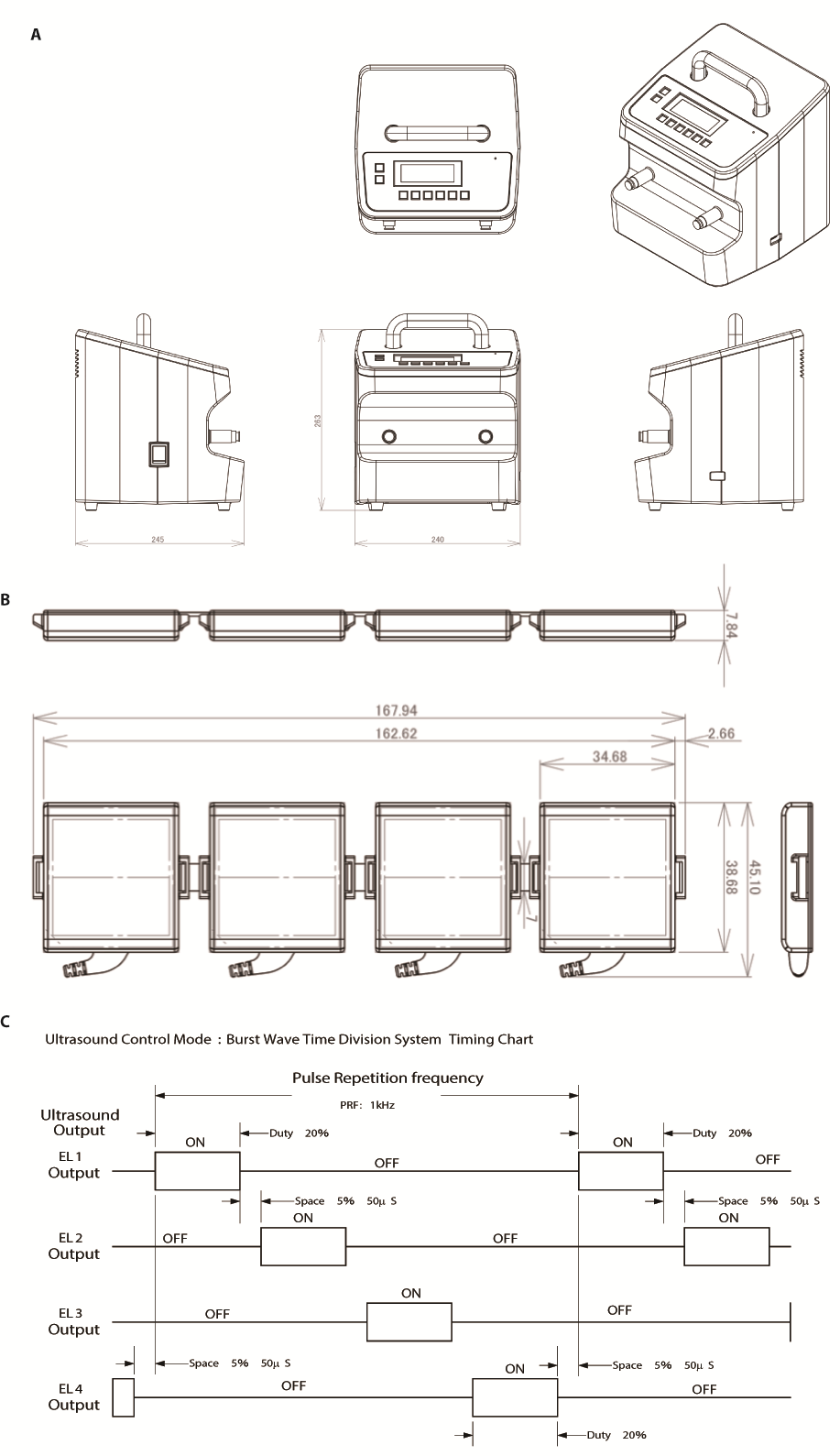


**Composition**

Main body

AC power cord

Transducer set: 2 sets (each set consists of 4 transducer elements or cells)

Ultrasonic gel

Fixing band: 2

**Specifications**

Main body size: width, 240 mm; depth, 245 mm; height, 263 mm

Weight: 5.5 kg

Power supply: 100 VAC~240VAC±10%

Electricity consumption: ≤ 150 VA

**Ultrasound output specifications**

Ultrasound transducer element size: width, 34.68 mm; depth, 38.68 mm; height, 7.84 mm

Ultrasound transducer element weight: 17.0 g

Ultrasound transducer element number: 8 elements (each transducer consists of 4 elements)

Ultrasound frequency: 2 MHz±10%

Ultrasound output power (ISATA): 30 mW/cm^2^

Beam non-uniformity ratio: 5±2

Pulse duration: 200 μs±5%

Pulse repetition frequency: 1 kHz±5%

Pulse duty: 20%±5%

Output duration: 20 minutes

**Supplemental Figure S1**


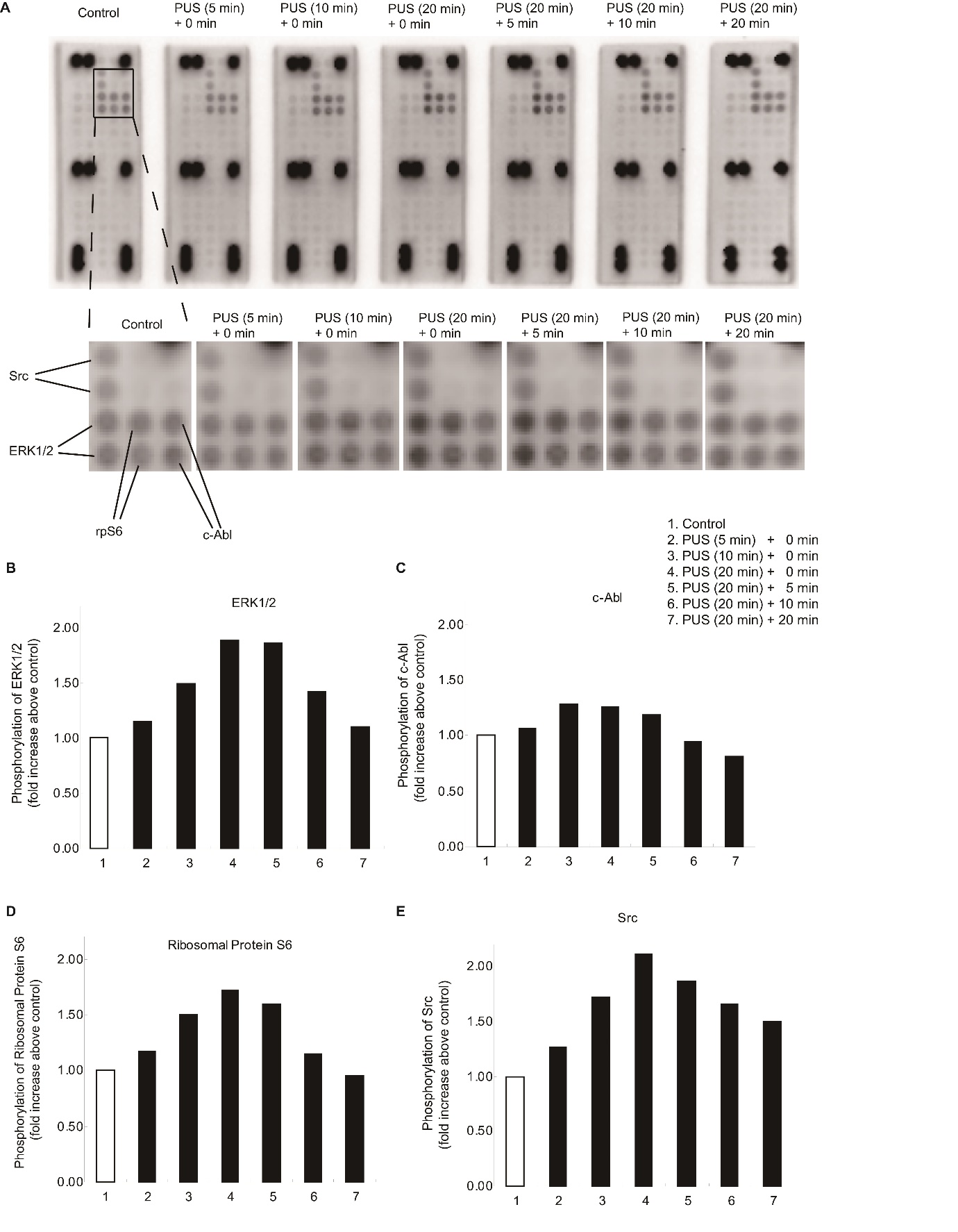


**Supplemental Figure S2**

**
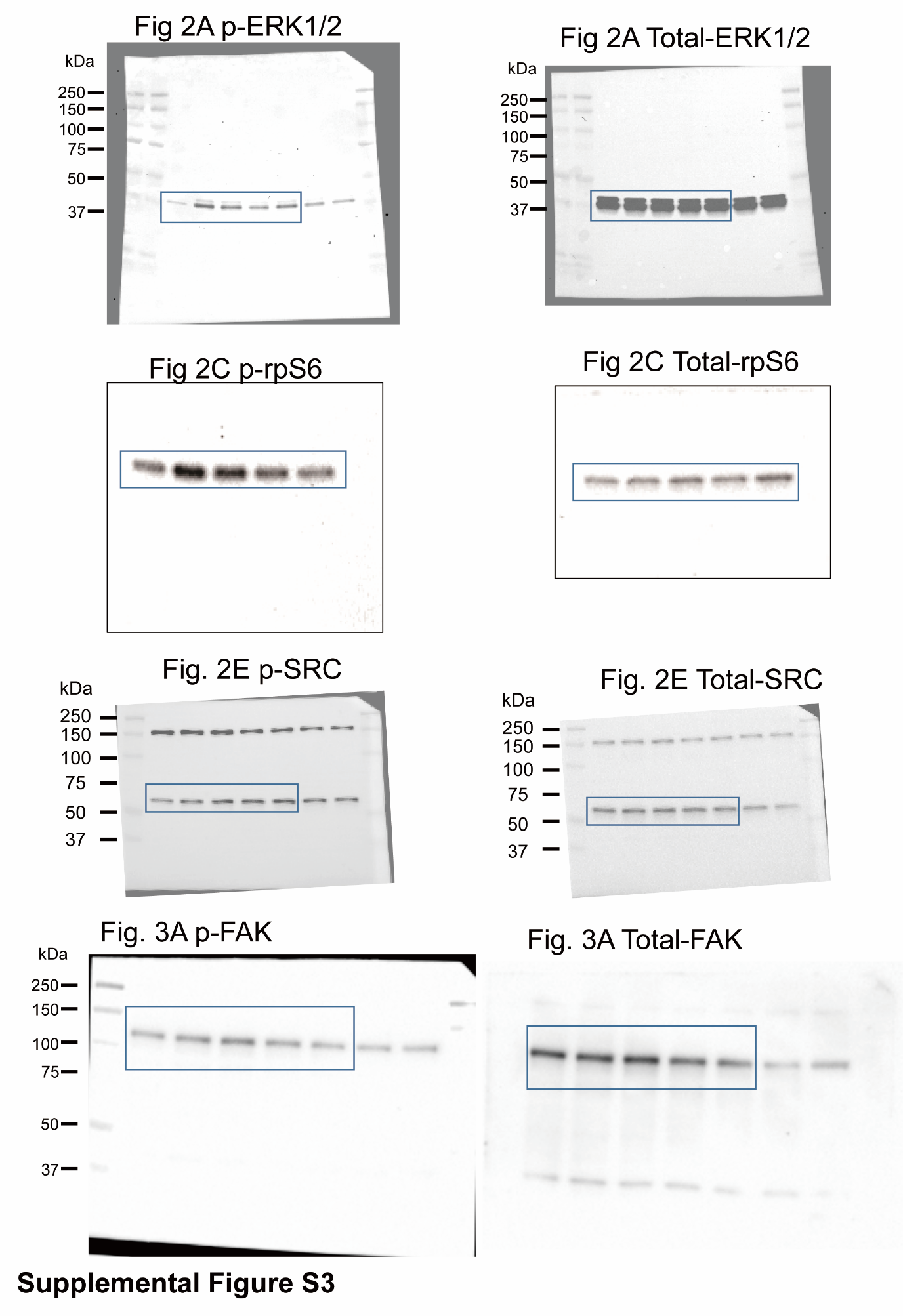
**
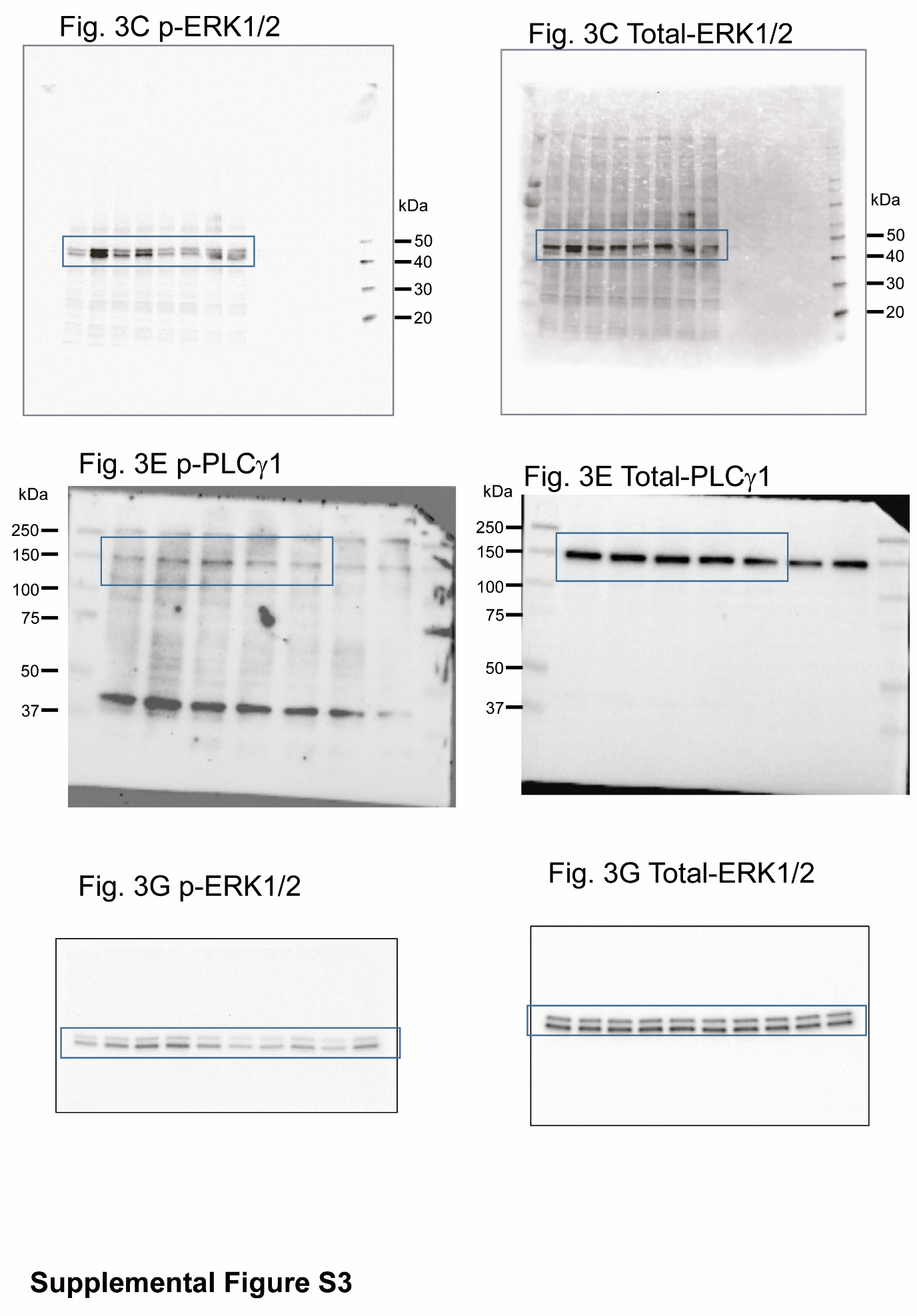

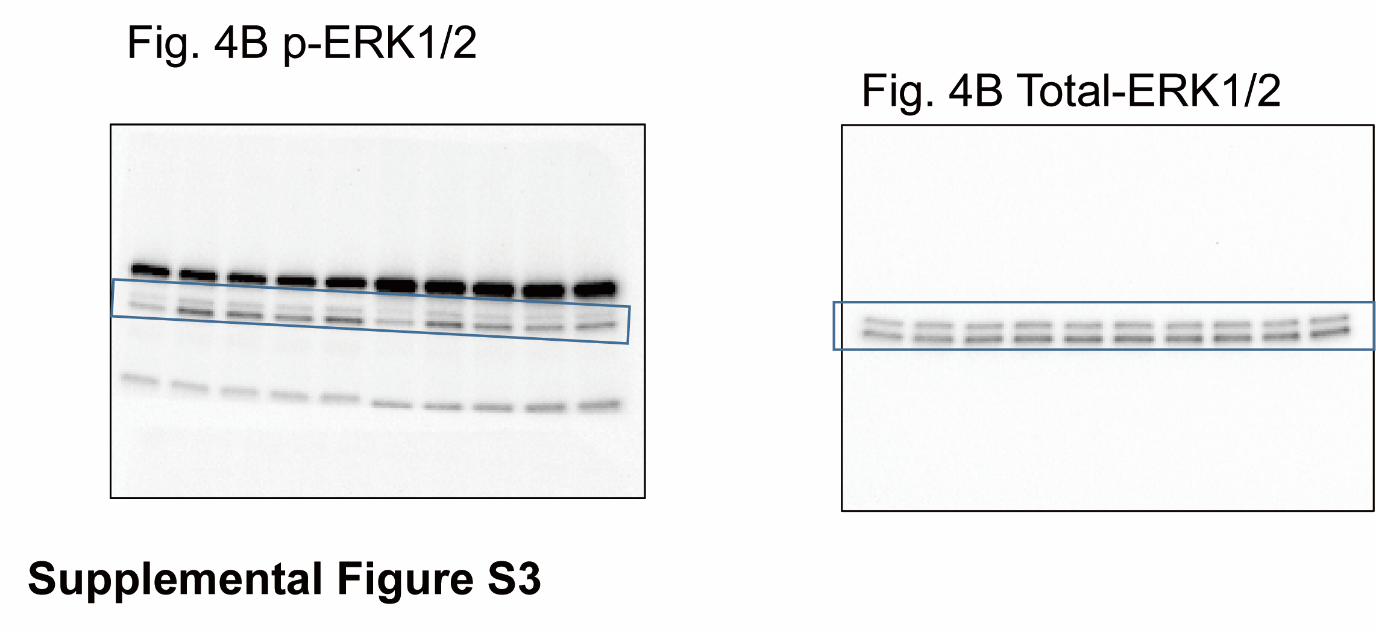

Supplement: Supplementary file 1 — Figures S1–S3. [file PHY2-14-e70718-s001.zip › Figure S1-S3.docx]
