# Supplementary figures and images for: Low‐intensity ultrasound induces angiogenesis by activating endothelial integrin signaling in male mice
Source: Physiol Rep. 2026 Jun 26;14(12):e70718. doi: 10.14814/phy2.70718 (PMC13305677; doi:10.14814/phy2.70718)

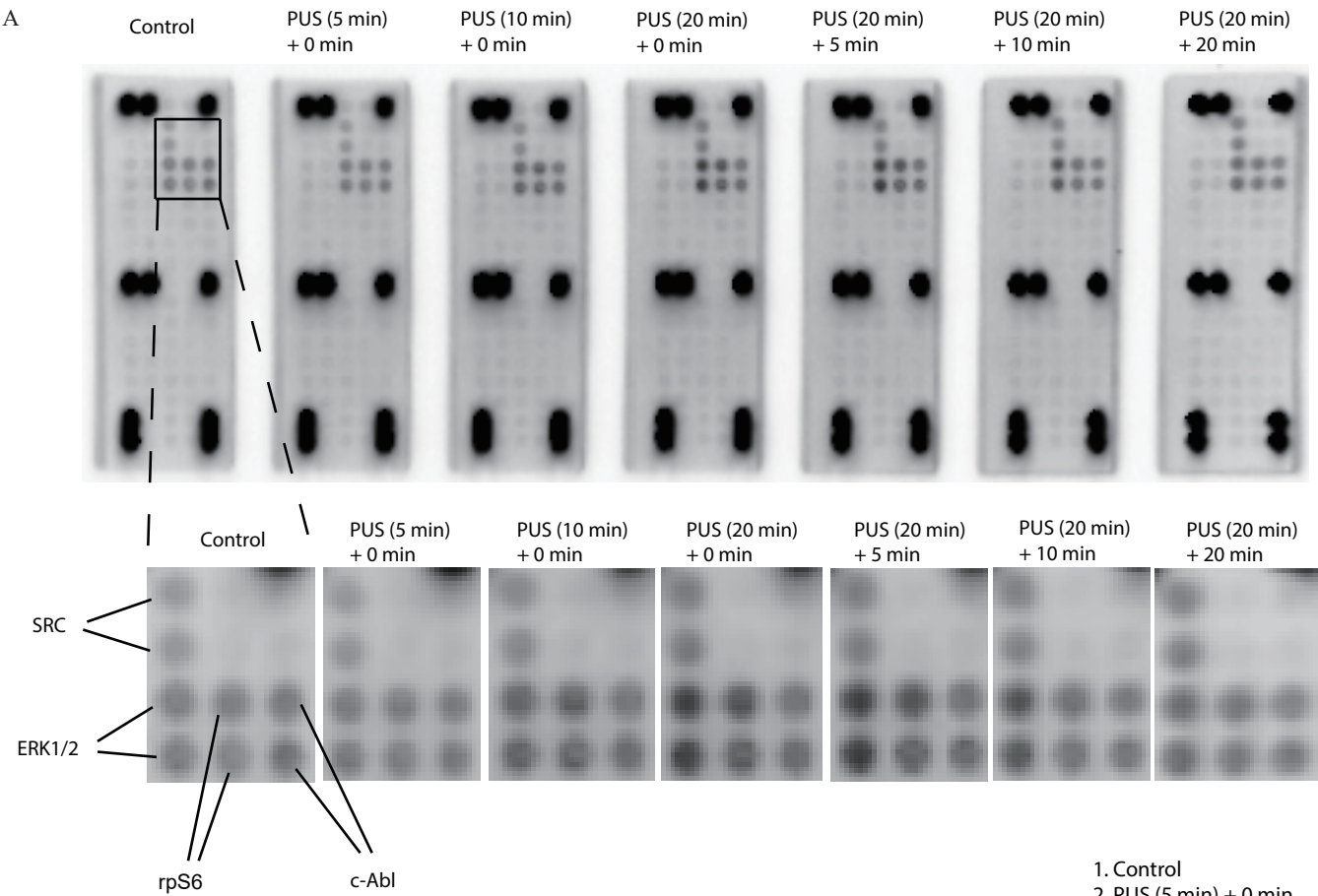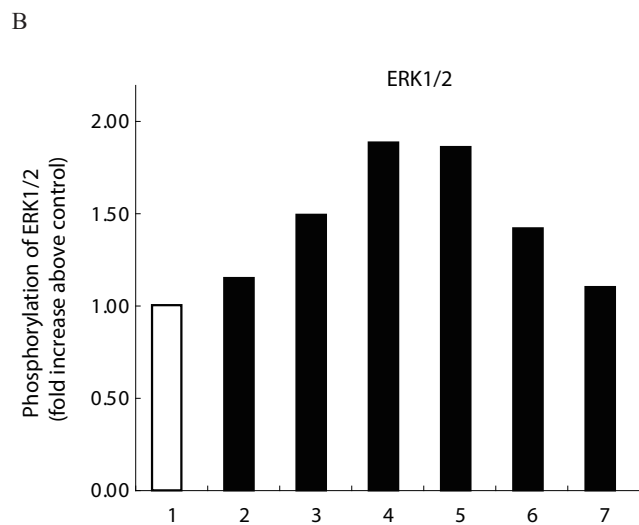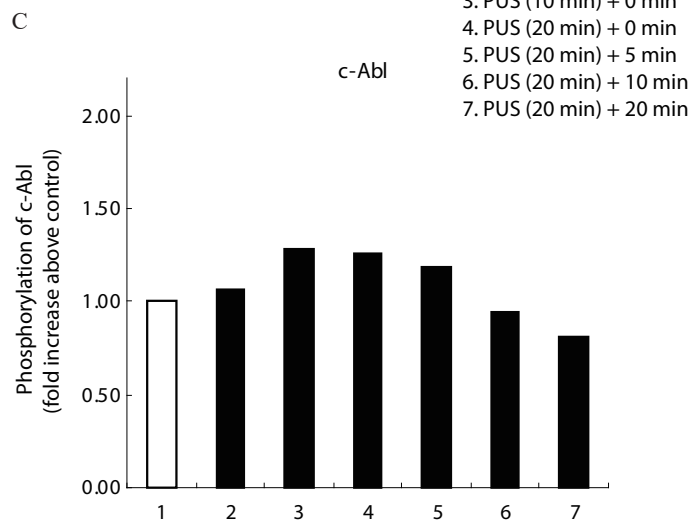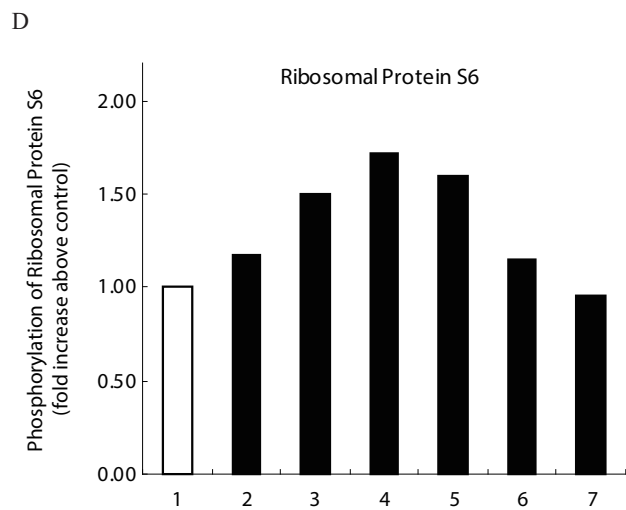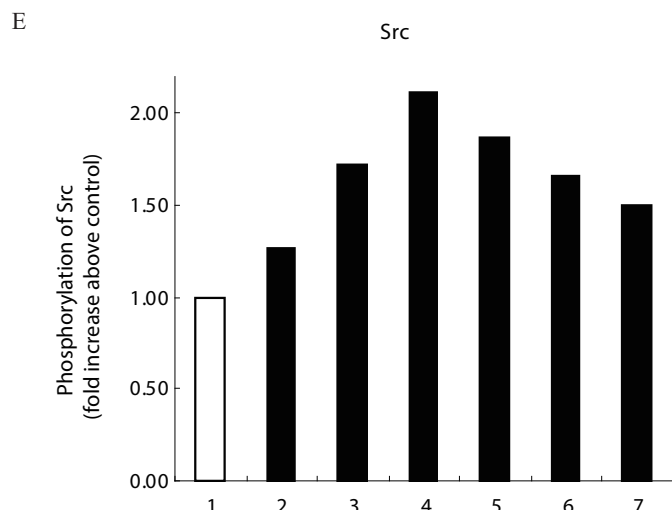

Supplement: Supplementary file 1 — Figures S1–S3. [file PHY2-14-e70718-s001.zip › PHYSREP-2025-07-605-T-s03.pdf]

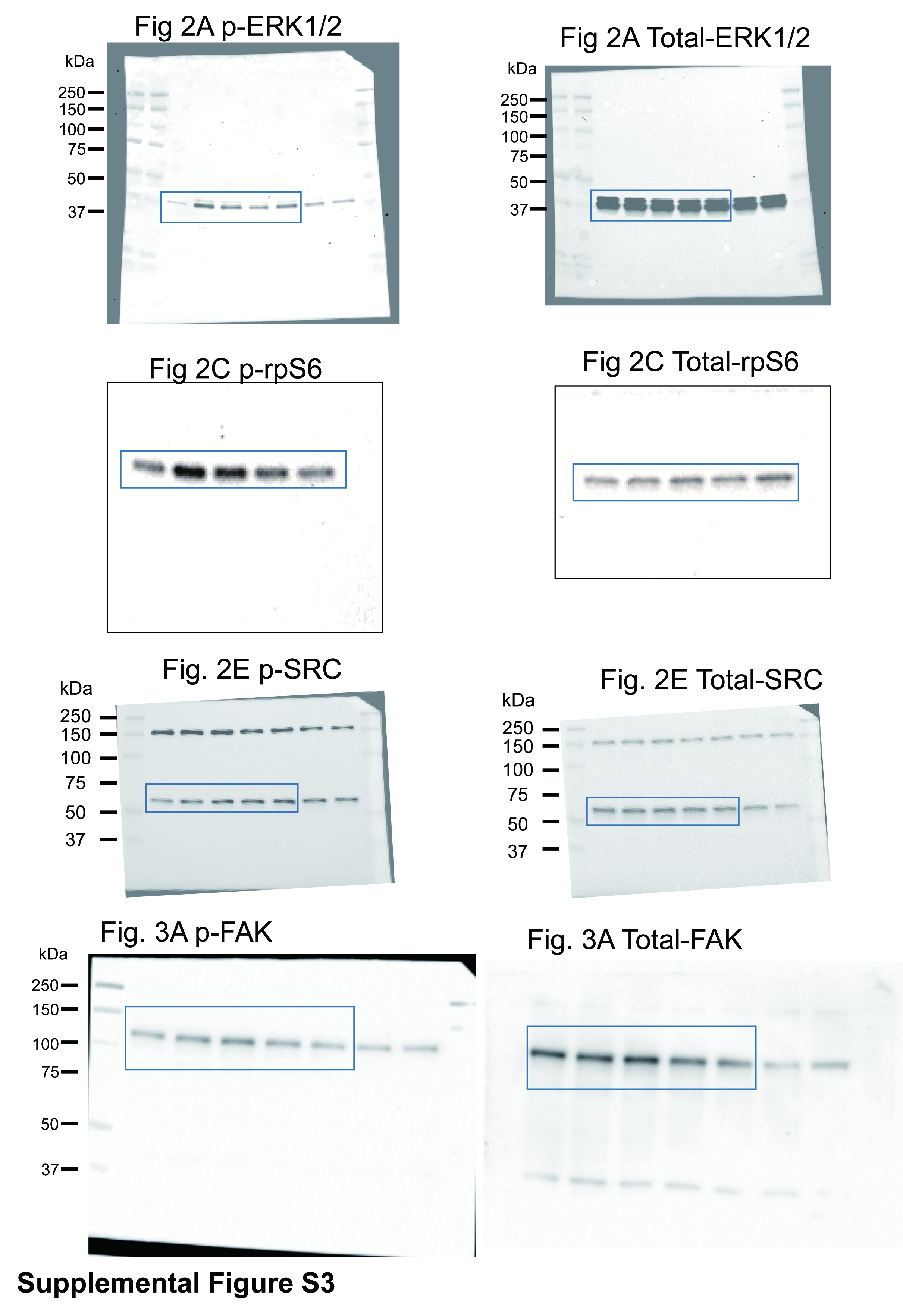

Supplement: Supplementary file 1 — Figures S1–S3. [file PHY2-14-e70718-s001.zip › PHYSREP-2025-07-605-T-s04.tif]

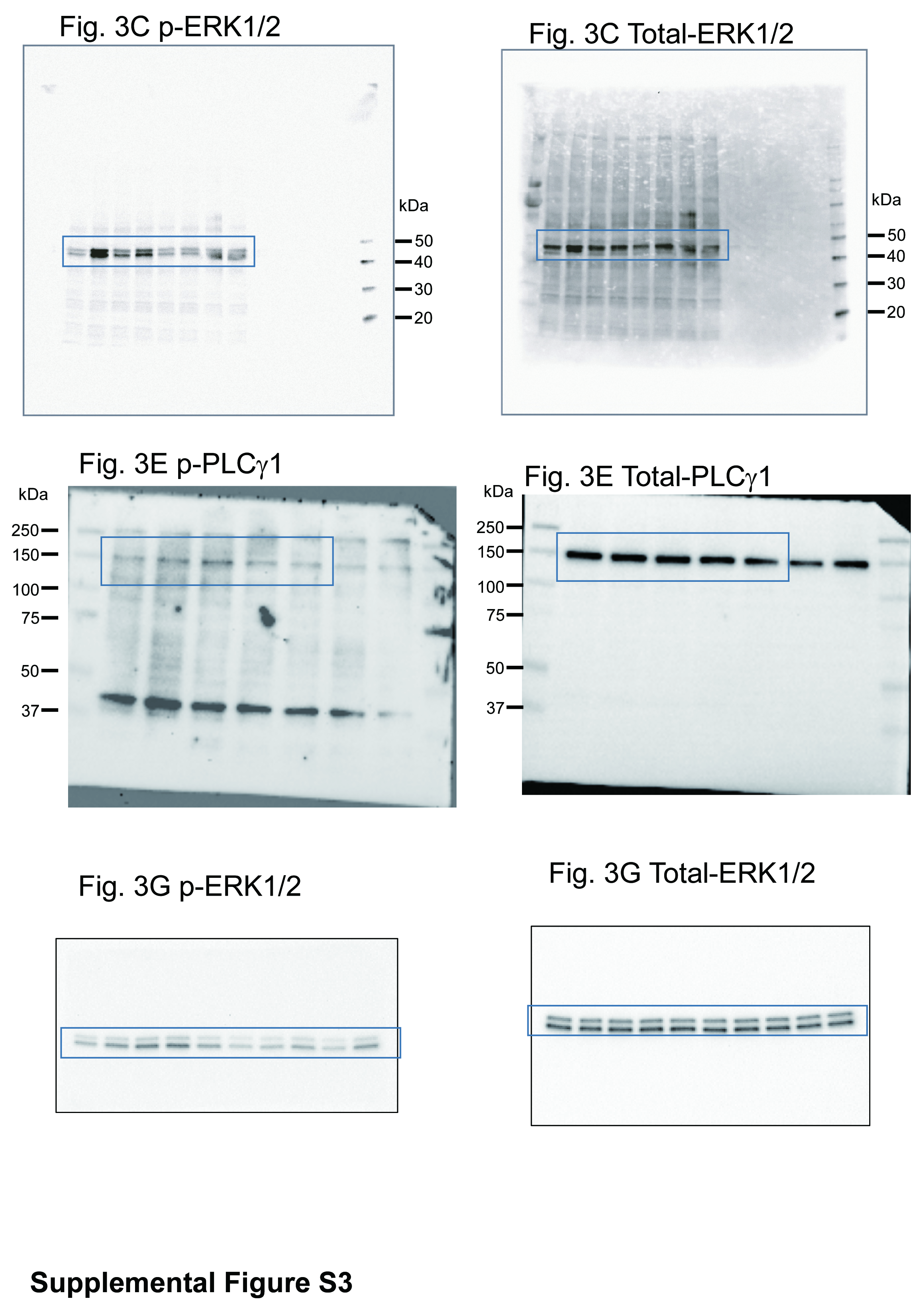

Supplement: Supplementary file 1 — Figures S1–S3. [file PHY2-14-e70718-s001.zip › PHYSREP-2025-07-605-T-s05.tif]

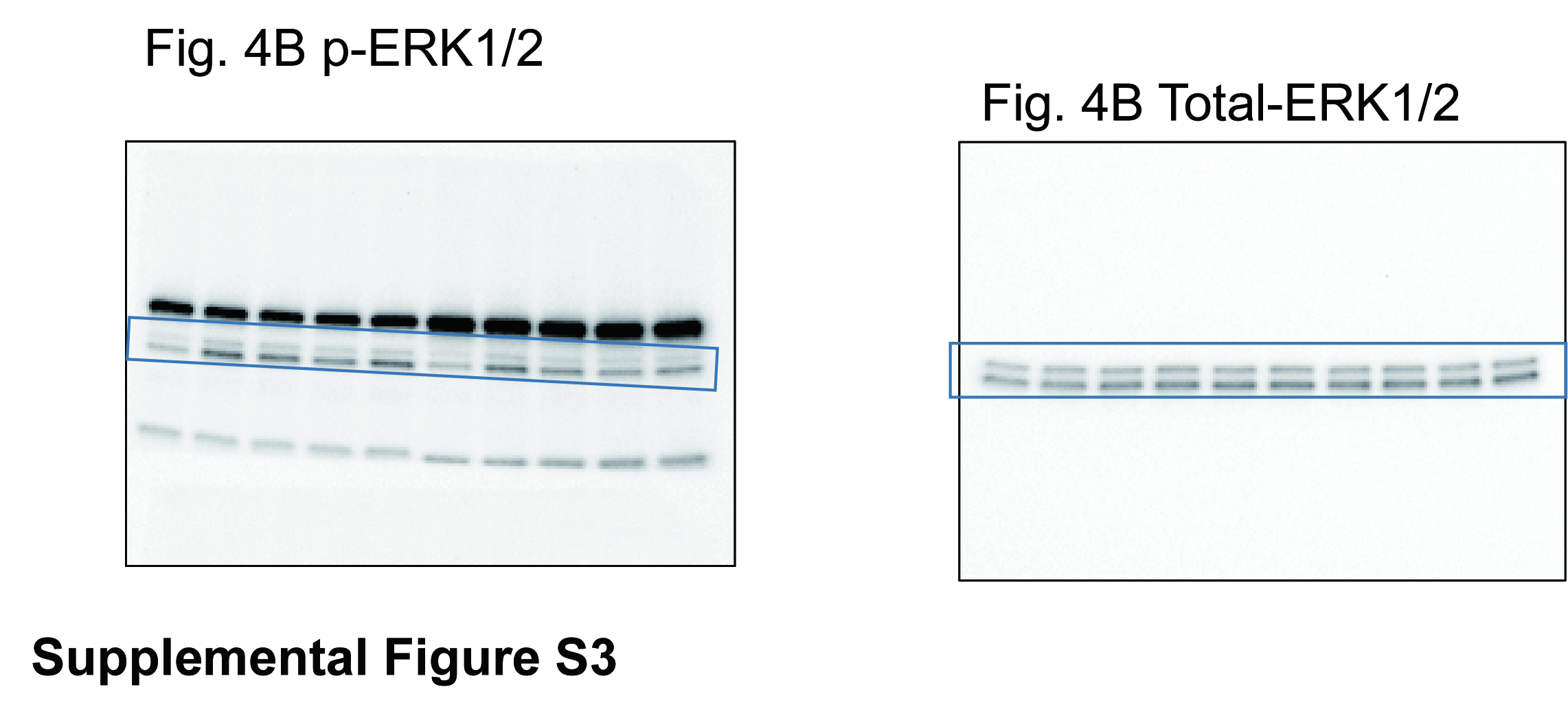

Supplement: Supplementary file 1 — Figures S1–S3. [file PHY2-14-e70718-s001.zip › PHYSREP-2025-07-605-T-s06.tif]
