# Supplementary material for: Low‐intensity ultrasound induces angiogenesis by activating endothelial integrin signaling in male mice
Source: Physiol Rep. 2026 Jun 26;14(12):e70718. doi: 10.14814/phy2.70718 (PMC13305677; doi:10.14814/phy2.70718)
Supplement: Supplementary file 2 — Table S1. [file PHY2-14-e70718-s002.docx]

Table S1. Antibodies used for immunoblotting

Antigen Supplier Antibody Conditions

**Immunohistochemical tissue staining**

CD31 #550274, BD Biosciences, USA 1:50

αSMA #A2547, Sigma-Aldrich, USA 1:50

**Tube formation assay**

β1 integrin-blocking antibody #ab7168, Abcam plc, UK 1.0 μg/mL

isotype-control IgG antibody #ab170190, Abcam plc, UK 1.0 μg/mL

**Western blotting**

total-ERK1/2 #4695, Cell signaling, USA 1:1,000

phospho-ERK1/2 (Thr202/Tyr204) #9106, Cell signaling, USA 1:2,000

total-Src #2109, Cell signaling, USA 1:2,000

phospho-Src (Tyr416) #6943, Cell signaling, USA 1:2,000

total-rpS6 #2217, Cell signaling, USA 1:1,000

phospho-rpS6 (Ser235/236) #4858, Cell signaling, USA 1:1,000

total-FAK #3285, Cell signaling, USA 1:2,000

phospho-FAK (Tyr397) #8556, Cell signaling, USA 1:2,000

total-PLCγ1 #2822, Cell signaling, USA 1:2,000

phospho-PLCγ1 (Tyr783) #2821, Cell signaling, USA 1:2,000

anti-Rabbit IgG HRP #NA934-1ML, GE Healthcare, UK 1: 10,000

**Immuno-Fluorescent assay**

integrin β1 #53550, Cell signaling, USA 1:300

anti-mouse IgG (Alexa488) #A11001, Thermo Fisher Scientific 1:700
